# Supplementary material for: An Andrographolide from Helichrysum caespitium (DC.) Sond. Ex Harv., (Asteraceae) and Its Antimicrobial, Antiquorum Sensing, and Antibiofilm Potentials
Source: Biology (Basel). 2021 Nov 24;10(12):1224. doi: 10.3390/biology10121224 (PMC8698270; doi:10.3390/biology10121224)
Supplement: Supplementary file 1 [file biology-10-01224-s001.zip › Figure S1 CF6 1H NMR.pdf]

Sample Name		Pulse sequence	PROTON	Temperature	25	Study owner	vnmr1
Date collected	2021-04-12	Solvent	cdcl3	Spectrometer	400MRpl-vnmrs400	Operator	vnmr1

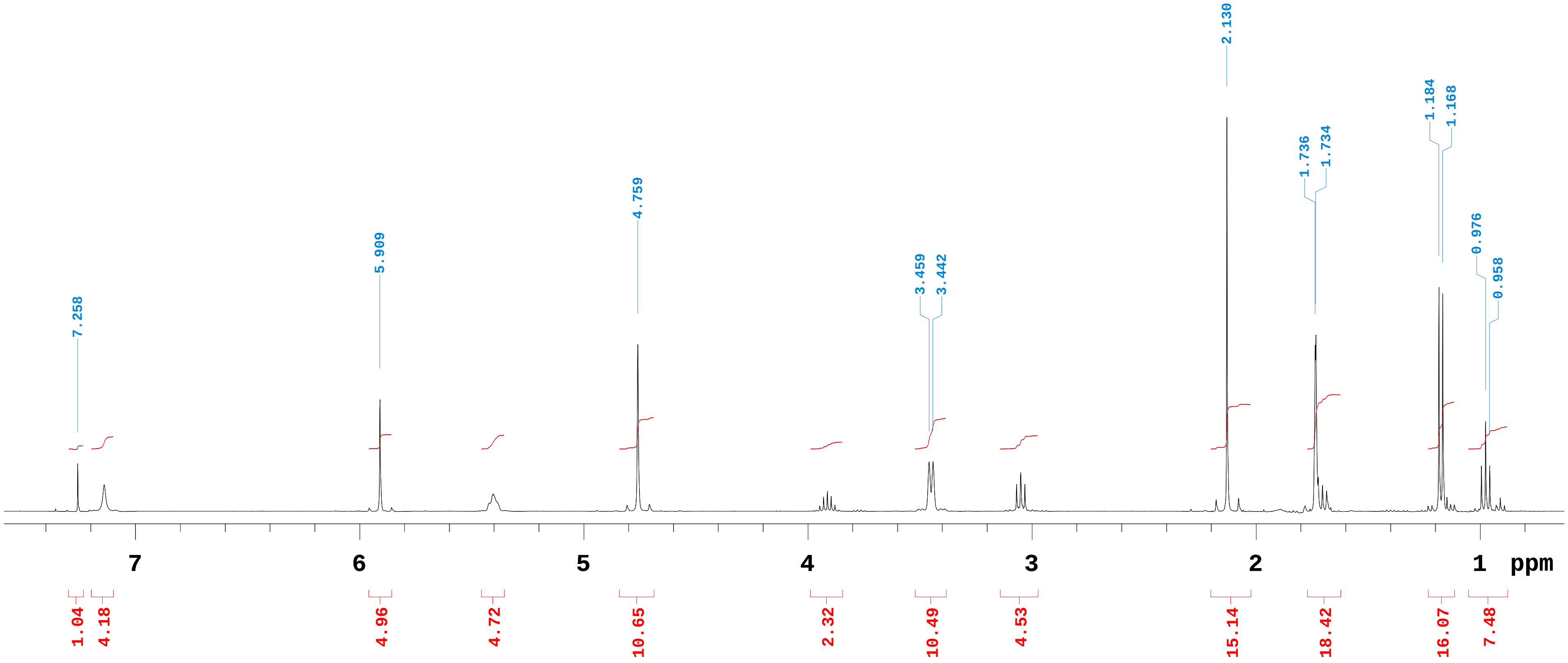

Data file /home/vnmr1/vnmrsys/data/Taki/TakiH2251b.fid

Plot date 2021-05-12

Sample Name		Pulse sequence	PROTON	Temperature	25	Study owner	vnmr1
Date collected	2021-04-12	Solvent	cdcl3	Spectrometer	400MRpl-vnmrs400	Operator	vnmr1

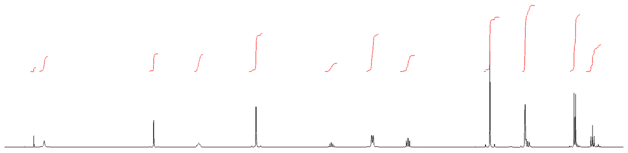

INTEGRAL VALUES

Integral	start(ppm)	end	value
1	7.29922	7.23249	1.035
2	7.19655	7.09902	4.180
3	5.95941	5.85675	4.963
4	5.45634	5.35368	4.718
5	4.84034	4.68634	10.651
6	3.9882	3.84447	2.315
7	3.52107	3.38247	10.489
8	3.1412	2.9718	4.534
9	2.20179	2.02213	15.141
10	1.77059	1.62172	18.422
11	1.23159	1.11352	16.074
12	1.05192	0.877387	7.478

Edward Bassey CF6

SAMPLE

date	Apr 12 2021
solvent	cdcl3
file	/home/vnmr1/vnmrsys /data/Taki/TakiH2251b.fid

ACQUISITION

sw	2976.2
at	2.753
np	16384
fb	4000
bs	32
d1	1.000
nt	8
ct	8

TRANSMITTER

tn	H1
sfrq	399.432
tof	-360.1
tpwr	59
pw	4.550

DECOUPLER

dn	C13
dof	0
dm	nnn
decwave	W40_HCN5mm
dpwr	38
dmf	29412

PRESATURATION

satmode	n
wet	n

SPECIAL

temp	not used
gain	36
spin	20
hst	0.008
pw90	9.100
alfa	10.000

FLAGS

il	n
in	n
dp	y
hs	nn

PROCESSING

fn	not used

DISPLAY

sp	250.0
wp	2780.4
rfl	-149.0
rfp	0
rp	-2.5
lp	0

PLOT

wc	252
sc	8
vs	70
th	7

ai cdc ph
