# Supplementary material for: An Andrographolide from Helichrysum caespitium (DC.) Sond. Ex Harv., (Asteraceae) and Its Antimicrobial, Antiquorum Sensing, and Antibiofilm Potentials
Source: Biology (Basel). 2021 Nov 24;10(12):1224. doi: 10.3390/biology10121224 (PMC8698270; doi:10.3390/biology10121224)
Supplement: Supplementary file 1 [file biology-10-01224-s001.zip › Figure S3 CF6-COSY.pdf]

Sample Name		Pulse sequence	**COSY**	Temperature	**25**	Study owner	**vnmr1**
Date collected	**2021-04-12**	Solvent	**cdcl3**	Spectrometer	**400MRpl-vnmrs400**	Operator	**vnmr1**

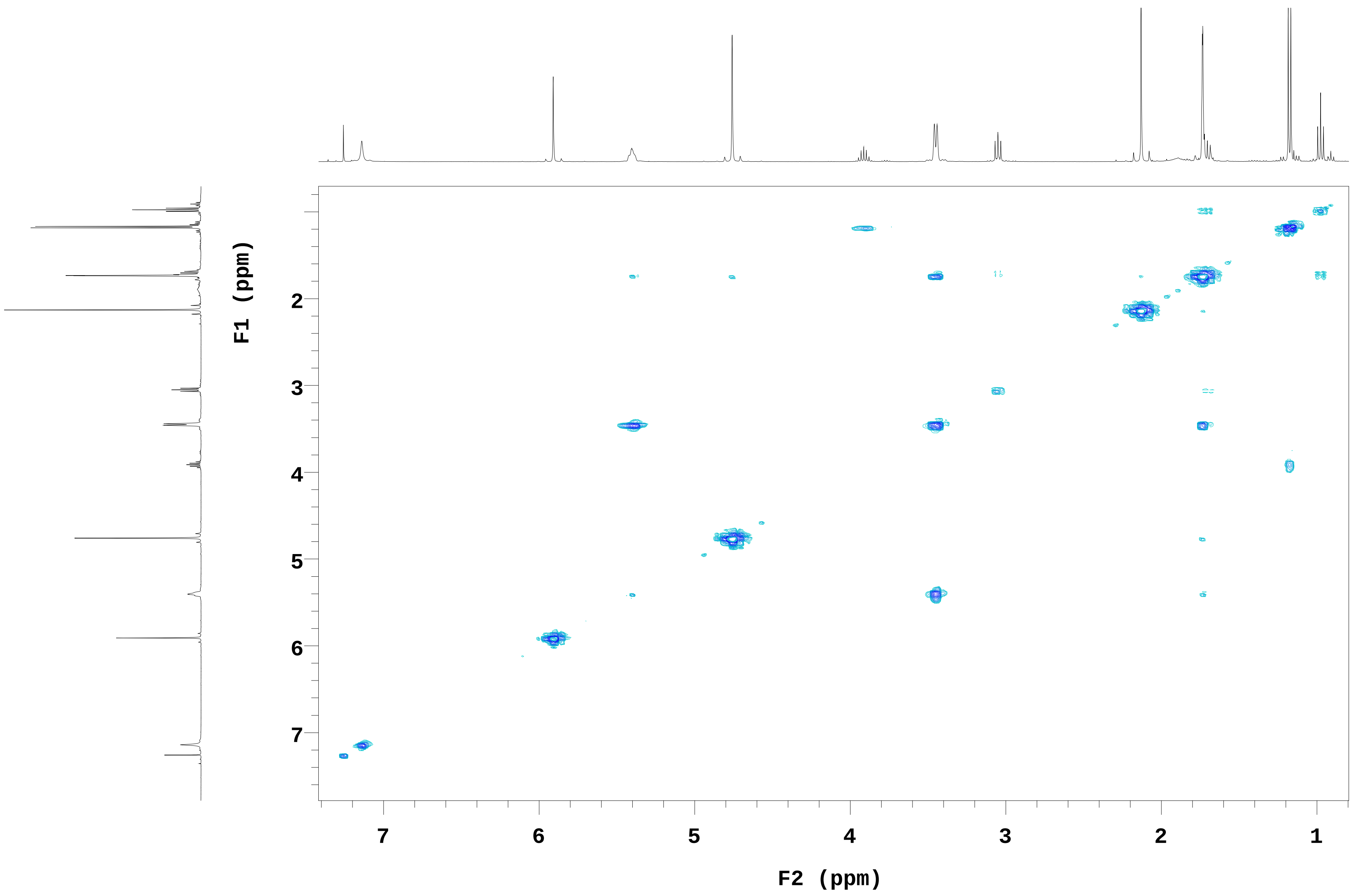

Data file /home/vnmr1/vnmrsys/data/Taki/TakiCOSY2251Hb.fid

Plot date 2021-05-13

Sample Name		Pulse sequence	COSY	Temperature	25	Study owner	vnmr1
Date collected	2021-04-12	Solvent	cdcl3	Spectrometer	400MRpl-vnmrs400	Operator	vnmr1

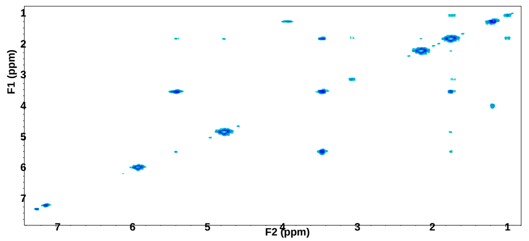

rfl1	-149.0
rfp1	0

PLOT

wc	257.4
sc	0
wc2	131.7
sc2	8.6
vs	167
th	6
ai cdc av	

Edward Bassey CF6

SAMPLE

date	Apr 12 2021
solvent	cdcl3
sample	

ACQUISITION

sw	2976.2
at	0.150
np	892
fb	4000
ss	32
d1	1.000
nt	2

2D ACQUISITION

sw1	2976.2
ni	128
d2	0

PRESATURATION

satmode	n
wet	n

TRANSMITTER

tn	H1
sfrq	399.432
tof	-360.1
tpwr	59
pw	9.100

GRADIENTS

PFGflg	y
hsglvl	1020
hsgt	0.0020

DECOUPLER

dn	C13
dm	nnn

FLAGS

hs	nn
sspul	y

SPECIAL

temp	not used
gain	36
spin	20

F2 PROCESSING

sb	-0.075
sbs	not used
fn	1024

F1 PROCESSING

sb1	-0.043
sbs1	not used
proc1	lp
fn1	1024

DISPLAY

sp	317.6
wp	2644.9
sp1	282.7
wp1	2825.1
rfl	-149.0
rfp	0

Data file /home/vnmr1/vnmrsys/data/Taki/TakiCOSY2251Hb.fid

Plot date 2021-05-13
