# Supplementary material for: An Andrographolide from Helichrysum caespitium (DC.) Sond. Ex Harv., (Asteraceae) and Its Antimicrobial, Antiquorum Sensing, and Antibiofilm Potentials
Source: Biology (Basel). 2021 Nov 24;10(12):1224. doi: 10.3390/biology10121224 (PMC8698270; doi:10.3390/biology10121224)
Supplement: Supplementary file 1 [file biology-10-01224-s001.zip › Figure S4 CF6 ATP.pdf]

Sample Name	Pulse sequence **APT**	Temperature **25**	Study owner **vnmr1**
Date collected **2021-05-13**	Solvent **cdcl3**	Spectrometer **400MRpl-vnmrs400**	Operator **vnmr1**

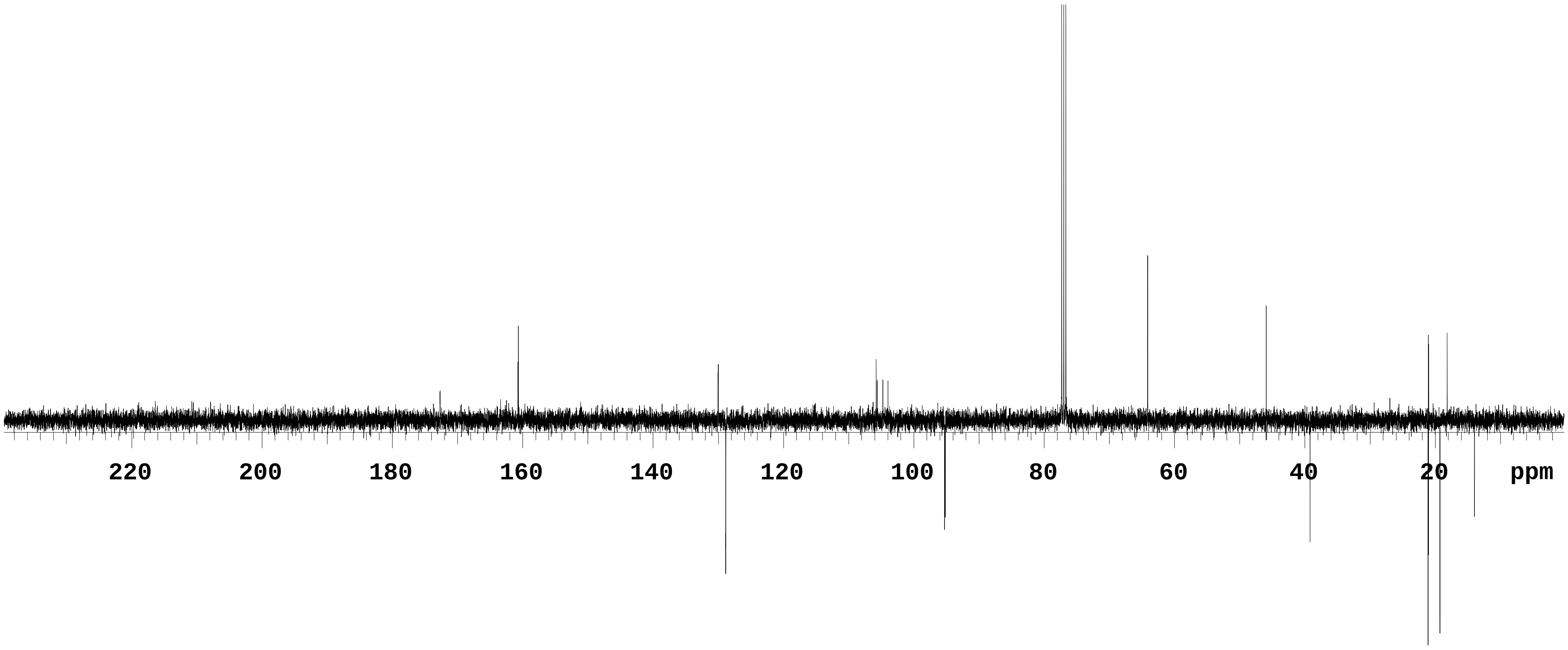

Sample Name		Pulse sequence	APT	Temperature	25	Study owner	vnmr1
Date collected	2021-05-13	Solvent	cdcl3	Spectrometer	400MRpl-vnmrs400	Operator	vnmr1

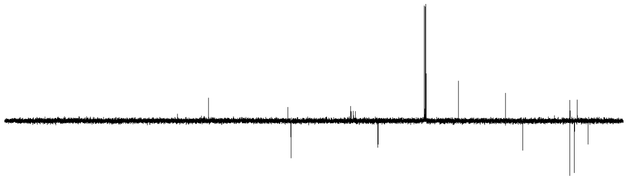

STANDARD PHOSPHORUS PARAMETERS

SAMPLE

date	May 13 2021
solvent	cdcl3
sample	

ACQUISITION

sw	24038.5
at	1.311
np	63016
bs	64
ss	4
d1	1.000
nt	512
ct	512

TRANSMITTER

tn	C13
tof	2523.7
tpwr	61
pw	3.600
pw90	7.200

DECOUPLER

dn	H1
dof	0
dpwr	39
dm	yny
decwave	w
dmf	11085

APT

j1xh	146.0
d3	0.0010

SPECIAL

temp	25.0
gain	50
spin	20

PROCESSING

lb	0.50
fn	65536

SPECTRUM

wp	24037.7
sp	23.9
rp	19.7
lp	0
ai cdc ph	

REFERENCE

rfl	-23.2
rfp	0

PLOT

wc	252
sc	8
vs	9260
hzmm	95.39
th	0
