# Supplementary material for: An Andrographolide from Helichrysum caespitium (DC.) Sond. Ex Harv., (Asteraceae) and Its Antimicrobial, Antiquorum Sensing, and Antibiofilm Potentials
Source: Biology (Basel). 2021 Nov 24;10(12):1224. doi: 10.3390/biology10121224 (PMC8698270; doi:10.3390/biology10121224)
Supplement: Supplementary file 1 [file biology-10-01224-s001.zip › Figure S5 CF6 HSQC.pdf]

Sample Name	Pulse sequence **HSQC**	Temperature **25**	Study owner **vnmr1**
Date collected **2021-04-12**	Solvent **cdcl3**	Spectrometer **400MRpl-vnmrs400**	Operator **vnmr1**

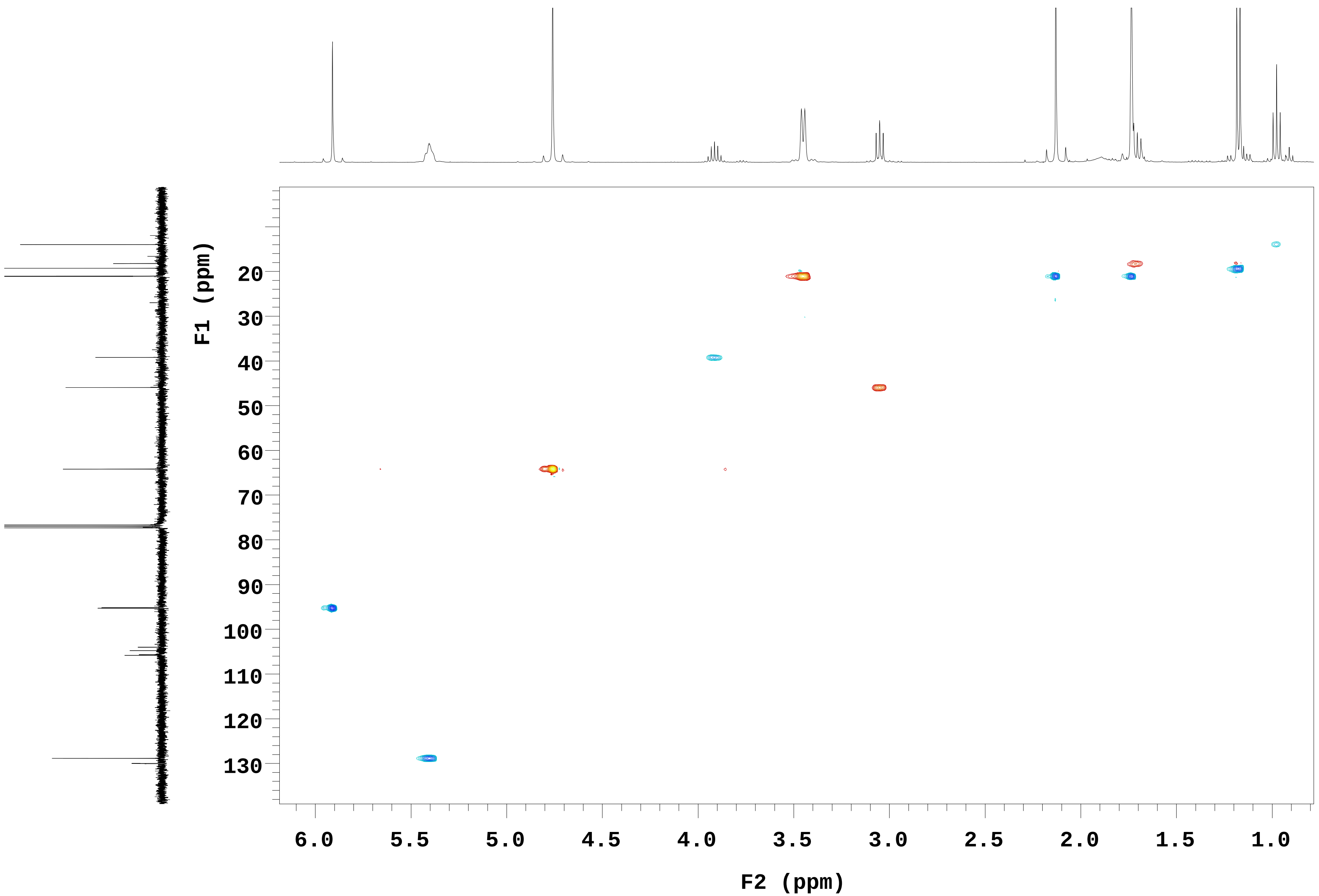

Data file /home/vnmr1/vnmrsys/data/Taki/TakiHSQC2251.fid

Plot date 2021-05-13

Sample Name		Pulse sequence	HSQC	Temperature	25	Study owner	vnmr1
Date collected	2021-04-12	Solvent	cdcl3	Spectrometer	400MRpl-vnmrs400	Operator	vnmr1

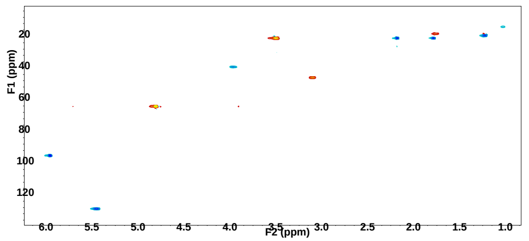

Edward Bassey CF6

SAMPLE

date	Apr 12 2021
solvent	cdcl3
sample	

ACQUISITION

sw	3453.0
at	0.150
np	1036
fb	4000
ss	32
d1	1.000
nt	4

2D ACQUISITION

sw1	24103.6
ni	128
phase	arrayed

TRANSMITTER

tn	H1
sfrq	399.432
tof	-170.4
tpwr	59
pw	9.100

DECOUPLER

dn	C13
dof	2533.7
dm	nny
decwave	W40_HCN5mm
dmf	29412
dpwr	38
pwxlvl	60
pwx	7.700

HSQC

j1xh	146.0
null	0
nullflg	y
mult	2

FLAGS

hs	nn
sspul	y
PFGflg	y
hsglvl	1020

SPECIAL

temp	not used
gain	36
spin	20

PRESATURATION

satmode	n
wet	n

F2 PROCESSING

gf	0.069
gfs	not used
fn	2048

F1 PROCESSING

gf1	0.007
gfs1	not used
proc1	lp
fn1	2048

DISPLAY

sp	312.7
wp	2158.1
sp1	118.3
wp1	13840.8
rfl	-100.3
rfp	0
rfl1	-0.6
rfp1	0

PLOT

wc	257.4
sc	0
wc2	131.7
sc2	8.6
vs	64
th	2
ai cdc ph	

ACQUISITION ARRAYS

array	phase
arraydim	256
i	phase
1	1
2	2

Data file /home/vnmr1/vnmrsys/data/Taki/TakiHSQC2251.fid

Plot date 2021-05-13
