# Supplementary material for: An Andrographolide from Helichrysum caespitium (DC.) Sond. Ex Harv., (Asteraceae) and Its Antimicrobial, Antiquorum Sensing, and Antibiofilm Potentials
Source: Biology (Basel). 2021 Nov 24;10(12):1224. doi: 10.3390/biology10121224 (PMC8698270; doi:10.3390/biology10121224)
Supplement: Supplementary file 1 [file biology-10-01224-s001.zip › Figure S6 CF6 gHMBC.pdf]

Sample Name		Pulse sequence	gHMBC	Temperature	25	Study owner	vnmr1
Date collected	2021-05-13	Solvent	cdcl3	Spectrometer	400MRpl-vnmrs400	Operator	vnmr1

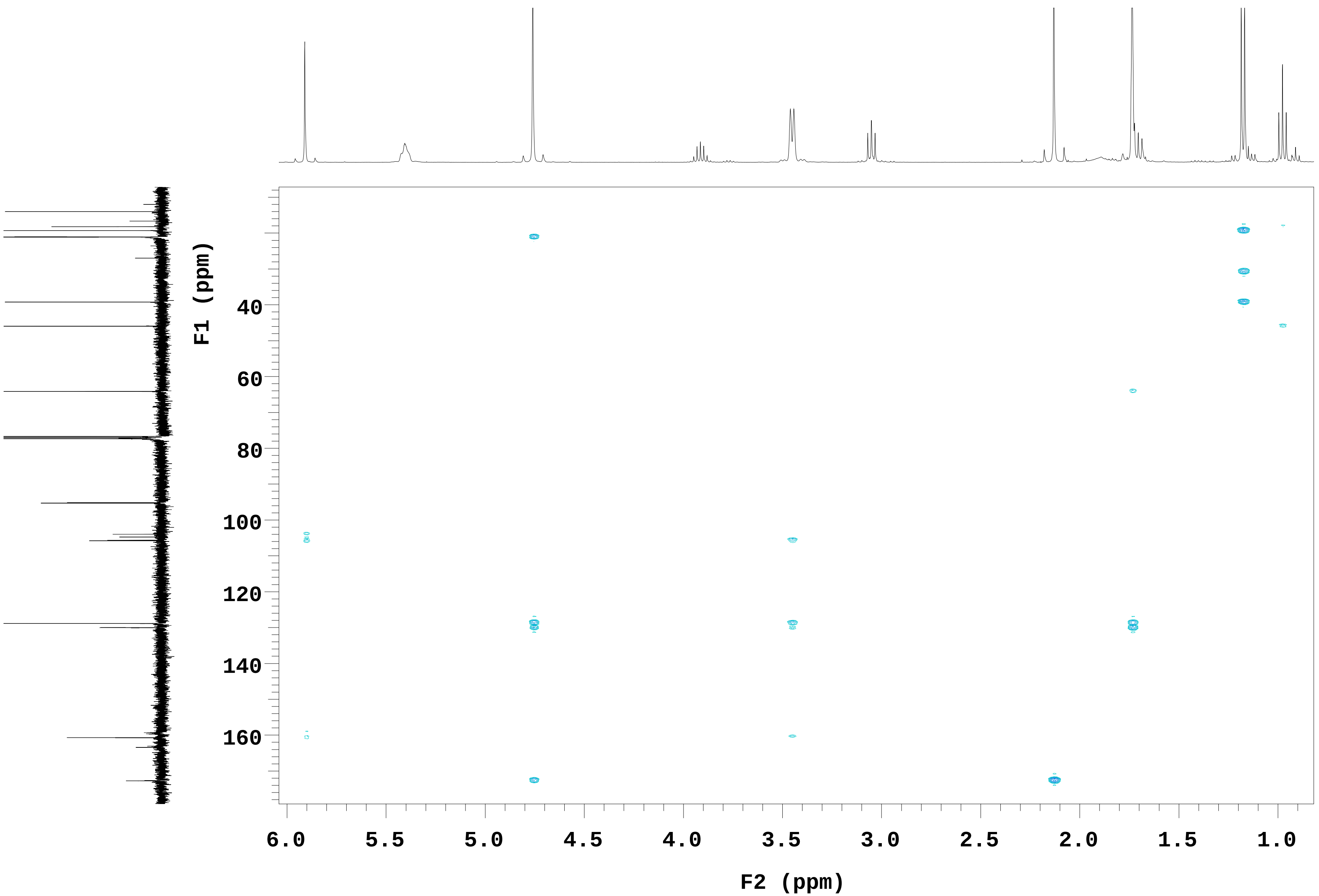

Sample Name		Pulse sequence	gHMBC	Temperature	25	Study owner	vnmr1
Date collected	2021-05-13	Solvent	cdcl3	Spectrometer	400MRpl-vnmrs400	Operator	vnmr1

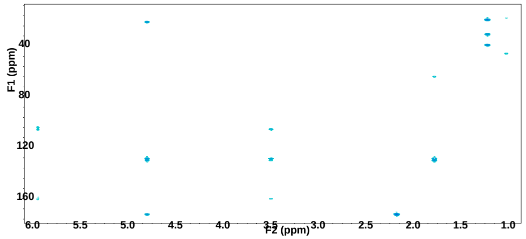

CF6

SAMPLE

date	May 13 2021
solvent	cdcl3
sample	

ACQUISITION

sw	2431.9
at	0.150
np	730
fb	4000
ss	32
d1	1.000
nt	8

2D ACQUISITION

sw1	18079.1
ni	256
phase	arrayed

PRESATURATION

satmode	n
wet	n

TRANSMITTER

tn	H1
sfrq	399.432
tof	-605.9
tpwr	59
pw	9.100

DECOUPLER

dn	C13
dof	-479.4
dm	nnn
decwave	W40_HCN5mm
dmf	29412
dpwr	38
pwxlvl	60
pwx	7.700

HMBC

j1xh	146.0
jnxh	8.0

FLAGS

hs	nn
sspul	y
PFGflg	y
hsglvl	1020

SPECIAL

temp	not used
gain	38
spin	20

GRADIENTS

gzlvl1	85
gt1	0.001000
gzlvl3	255
gt3	0.001000
gstab	0.000500

F2 PROCESSING

sb	-0.075
sbs	not used
fn	1024

F1 PROCESSING

gf1	0.013
gfs1	not used
proc1	lp
fn1	2048

DISPLAY

sp	327.3
wp	2085.2
sp1	723.6
wp1	17266.9
rfl	-175.3
rfp	0
rfl1	0.2
rfp1	0

PLOT

wc	257.4
sc	0
wc2	131.7
sc2	8.6
vs	167
th	3
ai cdc av	

ACQUISITION ARRAYS

array	phase
arraydim	512
i	phase
1	1
2	2
