# Supplementary figures and images for: An Andrographolide from Helichrysum caespitium (DC.) Sond. Ex Harv., (Asteraceae) and Its Antimicrobial, Antiquorum Sensing, and Antibiofilm Potentials
Source: Biology (Basel). 2021 Nov 24;10(12):1224. doi: 10.3390/biology10121224 (PMC8698270; doi:10.3390/biology10121224)

CARBON\_02

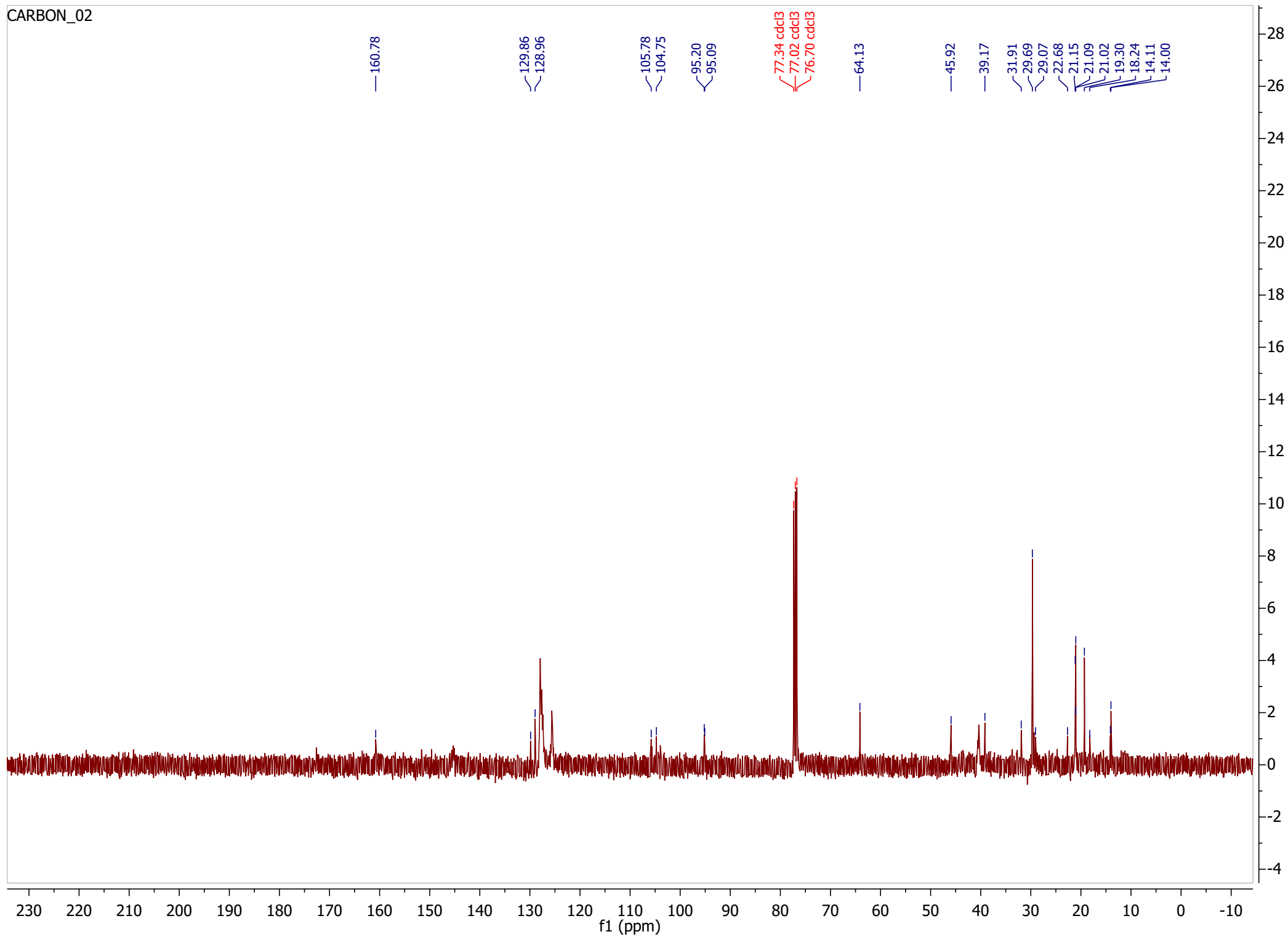

Supplement: Supplementary file 1 [file biology-10-01224-s001.zip › Figure S2 CF6 13C NMR.pdf]

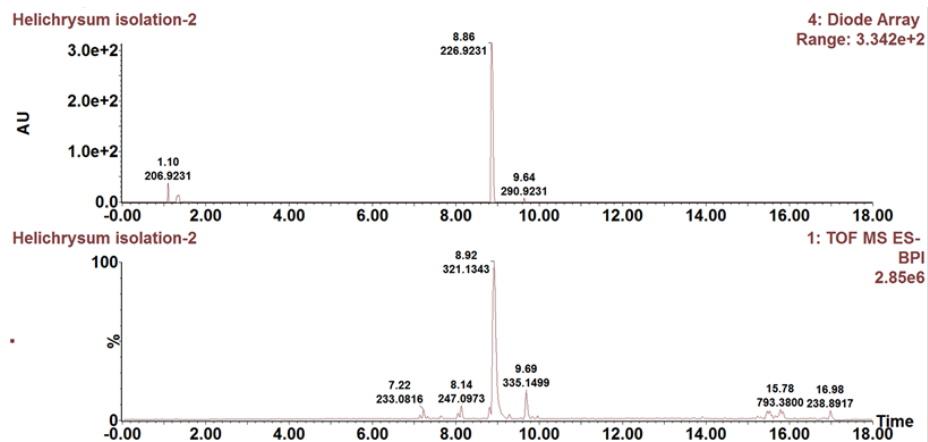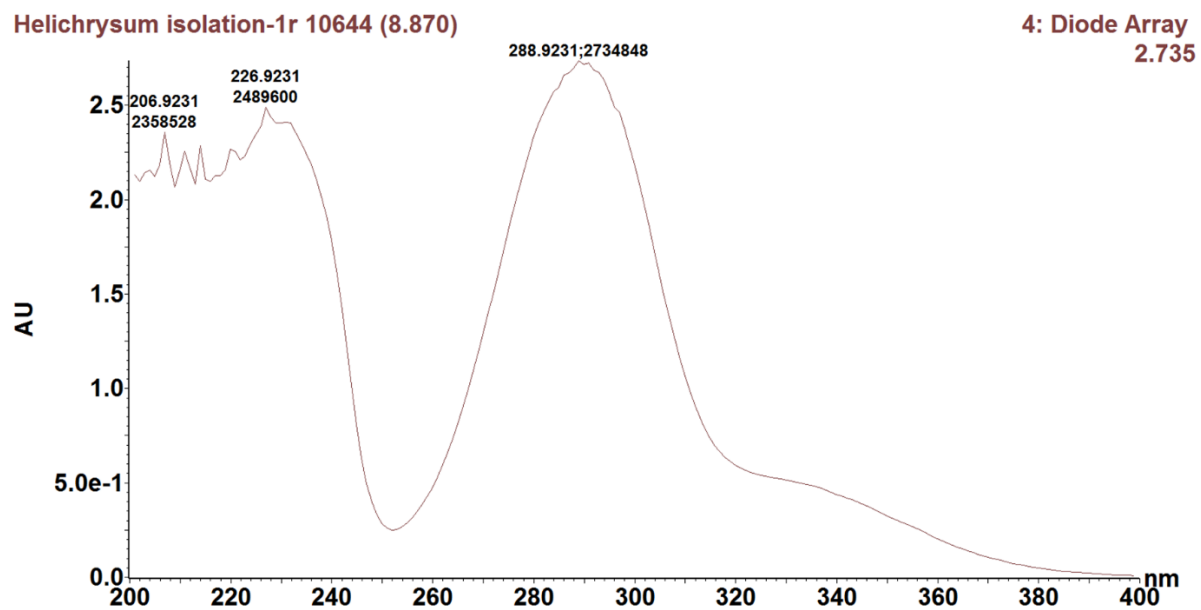

Helichrysum isolation-1 461 (8.939)

1: TOF MS ES-  
2.20e6

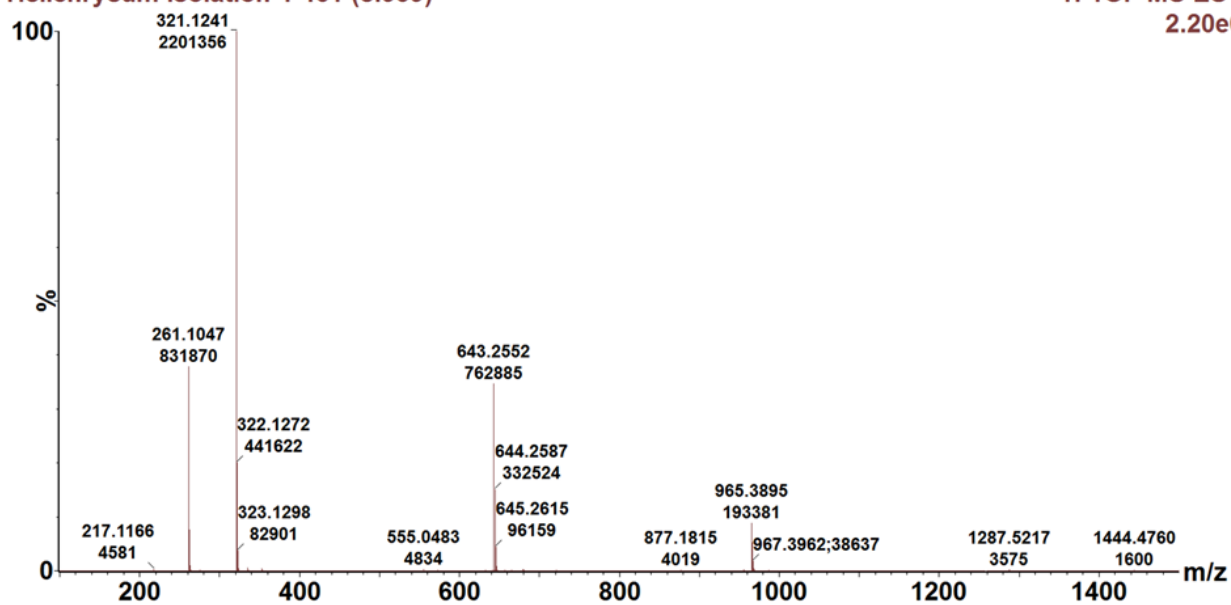

Supplement: Supplementary file 1 [file biology-10-01224-s001.zip › Figure S7 CF6 MASS DATA.pdf]
